# Younger vampire bats (*Desmodus rotundus*) are more likely than adults to explore novel objects

**DOI:** 10.1371/journal.pone.0196889

**Published:** 2018-05-03

**Authors:** Gerald G. Carter, Sofia Forss, Rachel A. Page, John M. Ratcliffe

**Affiliations:** 1 Department of Collective Behaviour, Max Planck Institute for Ornithology, Konstanz, Germany; 2 Department of Biology, University of Konstanz, Konstanz, Germany; 3 Group of Prehistory & Archaeology Science, University of Tübingen, Tübingen, Germany; 4 Smithsonian Tropical Research Institute, Balboa, Ancón, Panama; 5 Department of Biology, University of Toronto Mississauga, Mississauga, Ontario, Canada; University of Western Ontario, CANADA

## Abstract

The effects of age on neophobia and exploration are best described in birds and primates, and broader comparisons require reports from other taxa. Here we present data showing age-dependent exploration in a long-lived social species, the common vampire bat (*Desmodus rotundus*). A previous study found that vampire bats regurgitated food to partners trapped in a cage. Interestingly, while only a few adult bats visited the trapped bat, in every trial all or most of the eight young males in the colony would visit the trapped bat without feeding it. To test whether this behavioral difference resulted from age class differences in exploration, we compared responses of the bats to a trapped conspecific versus an inanimate novel object. Some adults and young showed interest in trapped conspecifics, but only the young males explored the novel objects. Additional novel object tests in a second captive colony showed that higher rates of novel object exploration were shown by young of both sexes. Our results corroborate past findings from other mammals and birds that age predicts exploration. If age-dependent exploration is indeed adaptive, then the role of age as a predictor of exploration tendency should depend on species-specific life history traits. Finally, because younger vampire bats also appear to have higher exposure to pathogens such as rabies virus, there may be implications for pathogen transmission if younger and more exploratory vampire bats are more likely to feed on novel hosts.

## Introduction

In many birds and mammals, immature individuals appear to be more exploratory and curious relative to their adult conspecifics [1–12]. Measuring responses to novel objects has been a simple and standard method of comparing the tendency for exploration between and within species [1–20]. However, exploration of novel objects has also been found to increase with age [12, 21]. One hypothesis that may reconcile these observations is that exploration is predicted to peak during a critical period in development, when individuals are old enough to be independent but are still assessing their environment through trial-and-error learning [22, 23].

If an early period of enhanced exploration increases opportunities for learning, this could be particularly adaptive for long-lived animals with flexible foraging strategies [24]. Most mammals appear to become proficient at foraging before their growth and reproductive development is complete. However, foraging proficiency is delayed and closer to the age of first reproduction for mammalian species with complex foraging strategies, slow development, post-weaning provisioning, high sociality, and sharing of food resources [24]. These traits have been argued as causal factors for the unusually delayed age for skill acquisition in humans relative to other primates [24].

Common vampire bats (*Desmodus rotundus*) also possess all these traits. They use flexible stalking tactics to feed upon the blood of diverse vertebrate hosts, and they are characterized by a long lifespan, slow reproduction, high parental investment, and long-term cooperative social relationships [25–29]. Individuals can survive for more than 29 years in captivity (GGC, personal observation) and more than 17 years in the wild [28]. The gestation period has been estimated at 5.5 to 7.3 months, and sexual maturity can take up to 9.5 months [29, 30]. Young vampire bats depend on mothers for longer than other bat species. Lactation periods for temperate and neotropical bats are typically 3–12 weeks, but female vampire bats nurse pups for 8–10 months [29, 31–36]. Regurgitated food sharing is important for young bats because individuals die after three consecutive unfed nights, and individuals younger than two years fail to feed on roughly one-third of nights, even when parasitizing abundant livestock [27]. Such age-dependent foraging success [37–39] is also consistent with a greater role for learning in mammals with slow development and food provisioning [24].

During a previous experiment [40], an isolated unfed bat was placed in a small cage within a larger flight cage housing a vampire bat colony, and food donors were found to regurgitate food across cage bars to a trapped recipient bat. We noticed that the trapped bat was also consistently visited by a group of eight young male bats that never provided food. The same eight young males would often visit collectively and also explore novel objects in the cage such as microphones and cables and would land and crawl upon people entering the cage. This increased boldness and exploration in young males could be due to the fact that males typically disperse from their natal colony between the age of 12–18 months [41, 42], or it could be due to a general effect of age. We define ‘young’ bats as those less than 18 months.

To determine if young males, or young bats in general, are more likely than older bats to explore novel objects, we conducted tests of novel object exploration first in the same captive group of vampire bats described above, which had eight young males and two young females, and then in a second mixed-age captive colony which had six young female and six young male vampire bats.

## Experiment 1

### Methods

This work was approved by the University of Maryland Institutional Animal Care and Use Committee (Protocol R-10–63). The first captive colony contained two young females, eight young males, 12 adult females, and 20 adult males. All individuals were born in captivity, and this colony is described in more detail elsewhere [40, 43, 44]. The eight young males were unmarked, and the other 34 bats had unique forearm band combinations. The fact that eight young males could not be individually distinguished did not lead to double-counting because we simply counted the maximum number of these bats seen simultaneously at any one time to get a conservative low-end estimate of how many unmarked bats visited. This approach worked because all eight unmarked bats often visited and explored the cage simultaneously (see below).

We exposed the captive bat colony to two different kinds of stimuli: (1) a conspecific groupmate trapped in a small cage and (2) an inanimate novel object. We first conducted 17 one-hour trials, during which we placed a bat inside a small cage on the floor of the larger flight cage and recorded infrared-illuminated video using a Sony Nightshot camcorder (see [40] for details). We counted a ‘visit’ as a bat approaching then touching the small cage holding the trapped bat (e.g. sniffing or crawling on it). We counted visits for the first 30 min of each of the 17 trials. To test whether bats were responding to novelty, we also exposed the colony to eight different novel inanimate objects, including the same cage but empty, and seven similarly-sized objects such as cardboard or plastic boxes. Again, we defined “visits” as a bat touching the novel object.

We counted the number of unique visitors as the response. To get a conservative minimum estimate of this response for the unmarked young males, we counted the greatest number of young males seen to visit the object together at one time in the trial (i.e. the minimum number of visitors in that trial). For example, if seven unmarked young males were observed crawling on the cage in one video frame, then the number of visiting young males was scored as seven. This approach is highly conservative because we should underestimate visits by young males, and we tested the alternative hypothesis of more visits by young males.

To test if young males were more likely than other bats to explore novel objects, and if they were more likely to visit trapped groupmates than novel inanimate objects, we first used Fisher’s exact test to compare the number of visits summed across all trials to each stimulus type (trapped bat or novel object) by category (young male or other). Second, we used permutation tests applied to generalized linear models (binomial distribution, logit link function) to test for the effect of stimulus type on the probability of visits by young males and by others. These tests compare the observed coefficients to those expected under the null hypothesis, simulated by randomizing the stimulus type across visits (5000 randomizations). To calculate 95% confidence intervals (CI) around the mean proportion of bats of each category that visited each stimulus type, we used nonparametric bootstrapping via the BCa method in the boot package in R [45, 46]. Data and R code are attached as a supplement (S1 Script).

### Results

**Fig 1:**
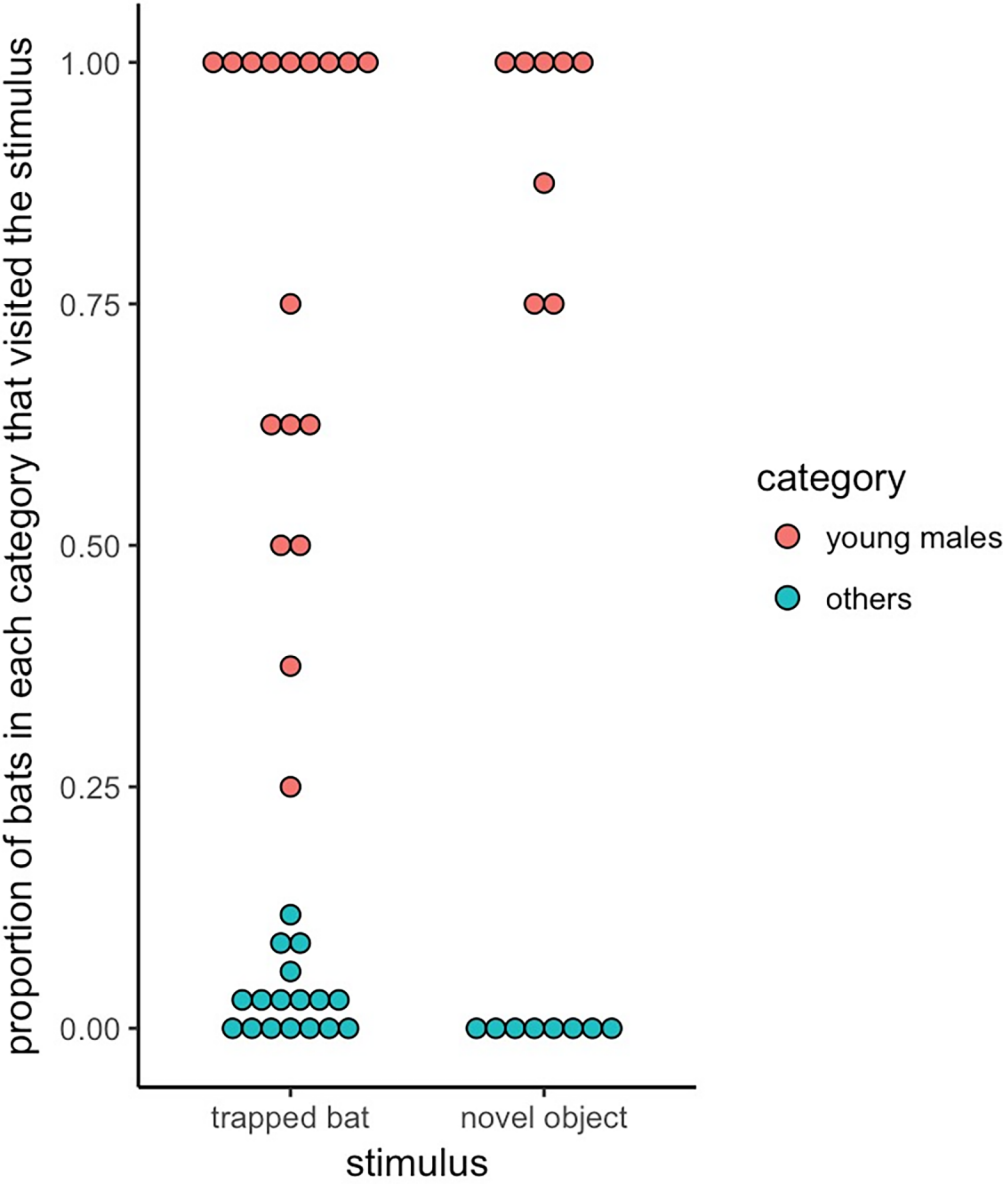
Young male vampire bats were more likely to visit trapped bats and novel objects compared to other bats. Dots of each color show responses in each trial. The lowest observed proportion of visiting young males was higher than the highest observed proportion of visiting bats that were female or adult male. Novel objects were always visited by more than five of the eight young males, whereas the other bats never visited a novel object.

The response towards trapped conspecifics versus novel objects depended on the category of bat. Most notably, all the observed visits to the novel objects were by the same eight young males. The 17 trapped bats resulted in at least 106 unique visits by the eight young males (six per trial) but only 18 unique visits from the 34 other bats (1 per trial), whereas the eight novel objects resulted in at least 59 unique visits by the young males (7 per trial) but zero visits from all other bats (Fisher’s exact test: odds ratio ~ 0, p < 0.001, permutation test interaction effect: p = 0.002, Fig 1). The young males did not visit trapped conspecifics significantly more or less than novel objects (permutation test, p = 0.1), but the other bats did (permutation test, p = 0.004). The minimum proportion of young males that visited did not differ between a trapped bat (mean = 0.78, 95% CI: 0.63–0.88) and a novel object (mean = 0.92, 95% CI: 0.81–0.97), but for the other bats, the proportion of visitors was greater for trapped bats (mean = 0.03, 95% CI: 0.02–0.05) than for novel objects (zero visits in 25 trials). We could not disentangle the effects of age and sex because only two young bats were female. We therefore performed more novel object presentations in a second captive group of bats sourced from a different wild population.

## Experiment 2

### Methods

This work was approved by the Smithsonian Tropical Research Institute Animal Care and Use Committee (#2015-0915-2018-A9), and by the Panamanian Ministry of the Environment (#SE/A-76-16). We worked with a second captive colony of 24 wild-caught female common vampire bats and their 12 captive-born offspring (six females, six males), which ranged in age from 3 to 15 months at the time of the tests. The colony had been housed for one year in a 1.7 x 2.1 x 2.3 m outdoor flight cage in Gamboa, Panama. All subjects were individually marked with visible unique forearm-bands.

From November 3 to 11, 2016, we conducted the first 12 trials starting at sunset (1750–1825 h) and then conducted eight additional trials from March 19 to April 12, 2017, later at night (2000 h). In each trial, a novel object roughly the size of a shoebox was placed on the flight cage floor for one hour and filmed with infrared illumination and a Sony Nightshot camcorder. Examples of novel objects were a 2-liter water bottle, a cardboard box, a tripod, and a rubber boot. The novel object and location was different for each trial. We used the statistical methods described above for experiment 1 to test if observed visits were disproportionately made by young bats, and to test for a difference in the mean proportion of bats that visited for each of three categories: young females, young males, and adult females.

### Results

**Fig 2:**
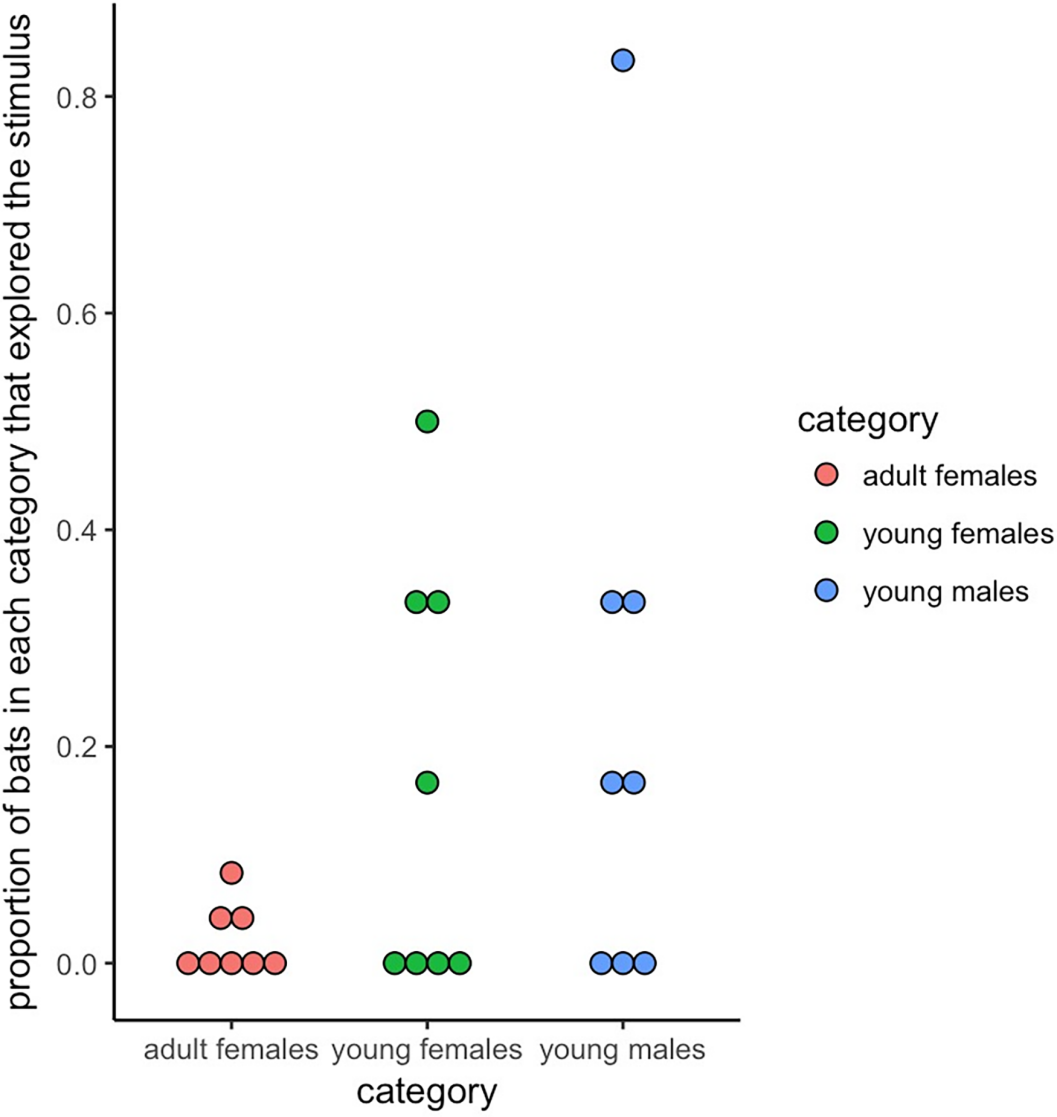
Young bats (six females and six males) visited novel objects more often compared to 24 adult females. Dots of each color show responses for trials in which the individual could be reliably identified. The majority of the proportions of visiting young bats were higher than the highest observed proportion of visiting adults.

We only analyzed visits in which bats could be reliably identified. In the first 12 trials conducted at sunset, we observed only a single visit, and the visiting bat could not be identified. In the next eight trials that we conducted later in the night at 2000 h, we could reliably identify the visiting bat in 23 of 54 total visits. Therefore, the visitation rate differed dramatically the first and second set of trials (permutation test: p = 0.005). In the second set, the identified visitors included two adult females, three young females, and three young males. As in experiment 1, age category influenced the proportion of bats that visited the novel object long enough to be identified; 19 of these 23 visits were by young bats (Fisher’s exact test: odds ratio = 0.09, p < 0.001; permutation test, p = 0.005, Fig 2), with 8 visits by young females and 11 visits by young males. The per capita probability of detecting a given individual visiting the novel object differed by age category (average visit probability for adult females: mean = 2%, 95% CI: 0–4%; for young bats: mean = 20%, 95% CI: 10–32%). There was not a large difference between the probability of visits by young females (mean = 17%, 95% CI = 4–29%) versus young males (mean = 23%, 95% CI = 8–46%). The young bats that visited the novel objects ranged in age from 7 to 12 months.

## Discussion

As previously demonstrated in other mammals and birds [1–12], we found that young common vampire bats were more likely to explore novel objects than adult conspecifics. In experiment 1, six to eight of the eight young males visited the novel objects compared to none of the 32 adults. The propensity for novel object exploration in young females was unclear due to an inadequate sample (n = 2). Young males could be especially exploratory because in the wild males typically disperse from their natal colony between 12–18 months of age, while young females are philopatric. Alternatively, young of both sexes could be more exploratory. In experiment 2, we confirmed our original age bias and found that female young bats were also more likely than adult females to visit novel objects.

By explicitly testing responses to novelty, our results also corroborate and extend previous evidence of age-dependent exploration in vampire bats [47]. Park [47] observed a captive colony of 15 common vampire bats that included three young males (3, 9, and 15 months old), two young females (2 and 10 months old) and noted that younger bats were more likely to engage in behavior interpreted as “play” and to explore inanimate objects. Exploration behavior was observed in the 2-month-old bat, increased with age until a peak in the two bats that were 9–10 months old, then diminished with age until adulthood [47].

Taken together, these observations suggest that, in comparison to adults, both young male and young female common vampire bats are more exploratory than are adult vampire bats of either sex. The age difference in our results corroborates other observations that young mammals might be more inclined to explore novel objects and situations as they are learning about their environment. In some species, immatures or adolescents peak in explorative behavior compared to younger infants and older adults [48–51], and exploration tendency might increase as dependence on maternal care ends. It might appear that the age-dependent effects of exploration we observed could have resulted from age-dependent food motivation, but we find this unlikely because fed bats also show the same apparent age difference in boldness and exploration. Even after feeding, the eight young males in experiment 1 were also likely to explore novel objects and to land and crawl on humans (GGC, personal observation).

Age-dependent “neophobia” might result from mechanisms other than fear. In experiment 1, adults responded to trapped conspecifics and not novel objects, but it is unclear whether this difference is due to greater fear or less interest in the inanimate objects. More experienced adults might simply be faster to classify a novel object as irrelevant or uninteresting, rather than dangerous [52].

The social environment is also likely to influence reactions to novelty because risk is reduced in groups. In social species, such as vampire bats, younger individuals are expected to use social cues for when and what to explore in their environment [53]. Bats in our study typically approached the novel objects together and it is likely that exploration rates were increased by bats being drawn to the presence of other individuals (i.e. local enhancement) [54]. If young bats attend more to the actions of their similar-aged peers, then age differences in exploration could be exaggerated [53]. To disentangle these effects, further experiments are needed that test exploration by individuals both alone and in groups.

In birds, dietary specialists are reported to be less exploratory and more neophobic, compared to opportunistic foragers that require increased behavioral flexibility [55, 56]. It is unclear whether to classify vampire bats as dietary specialists or opportunistically flexible foragers. On one hand, vampire bats drink only blood and this specialization has led to numerous adaptive changes in morphology [57], physiology [58], microbiome [59], and even cognition—specifically a loss of taste aversion learning [60]. On the other hand, they are capable of using different approach strategies to feed on a diversity of hosts [57, 61–64]. Today, the primary hosts of most vampire bats are livestock that were introduced only half a century ago.

Novel object exploration rates can vary with time of day and with situational risk [55, 65, 66]. The first set of 12 trials in experiment 2 yielded only a single visit, probably because they were conducted too early in the night. Vampire bats are often not active until late in the night when their hosts and potential predators are less active and able to detect them. Captive environments are clearly less risky, and captive individuals are often less neophobic than their wild counterparts [19, 67–69]. Our observations were likely to be influenced by captivity and the lack of negative reinforcement. Given the roles of age and situation in influencing exploration proclivity, it would be interesting to compare the influences of these factors in exploration of novel objects across species with different foraging strategies using both captive and wild populations. If age-dependent exploration is indeed adaptive, then the role of age as a predictor of exploration tendency should be greater in species where young individuals acquire some foraging skills through learning.

Finally, it would be interesting to test if younger vampire bats are more likely to feed on host species that are unfamiliar to them or that are in novel locations. If younger vampire bats are more likely than adults to feed on atypical hosts, such as dogs or humans, rather than cattle, this could have implications for pathogen spillover because younger vampire bats appear to experience higher exposure to pathogens such as rabies virus [70] and hemotropic *Mycoplasm*a [71].

## Ethics

Experiments were approved by the Smithsonian Tropical Research Institute Animal Care and Use Committee (#2015-0915-2018-A9) and by the Panamanian Ministry of the Environment (#SE/A-76-16).

## Supporting information

S1 Script Code and embedded data for reproducing analyses and plots. (R)
